# Microfluidic Sensors for Micropollutant Detection in Environmental Matrices: Recent Advances and Prospects

**DOI:** 10.3390/bios15080474

**Published:** 2025-07-22

**Authors:** Mohamed A. A. Abdelhamid, Mi-Ran Ki, Hyo Jik Yoon, Seung Pil Pack

**Affiliations:** 1 Faculty of Education and Arts, Sohar University, Sohar 311, Oman; mabdelhamid@su.edu.om; 2 Department of Biotechnology and Bioinformatics, Korea University, Sejong-ro 2511, Sejong 30019, Republic of Korea; allheart@korea.ac.kr; 3 Institute of Industrial Technology, Korea University, Sejong-ro 2511, Sejong 30019, Republic of Korea; 4 Institute of Natural Science, Korea University, Sejong-ro 2511, Sejong 30019, Republic of Korea; hyojik88@korea.ac.kr

**Keywords:** microfluidic sensors, micropollutants, environmental monitoring, lab-on-a-chip, point-of-care diagnostics, artificial intelligence, machine learning, nanomaterials

## Abstract

The widespread and persistent occurrence of micropollutants—such as pesticides, pharmaceuticals, heavy metals, personal care products, microplastics, and per- and polyfluoroalkyl substances (PFAS)—has emerged as a critical environmental and public health concern, necessitating the development of highly sensitive, selective, and field-deployable detection technologies. Microfluidic sensors, including biosensors, have gained prominence as versatile and transformative tools for real-time environmental monitoring, enabling precise and rapid detection of trace-level contaminants in complex environmental matrices. Their miniaturized design, low reagent consumption, and compatibility with portable and smartphone-assisted platforms make them particularly suited for on-site applications. Recent breakthroughs in nanomaterials, synthetic recognition elements (e.g., aptamers and molecularly imprinted polymers), and enzyme-free detection strategies have significantly enhanced the performance of these biosensors in terms of sensitivity, specificity, and multiplexing capabilities. Moreover, the integration of artificial intelligence (AI) and machine learning algorithms into microfluidic platforms has opened new frontiers in data analysis, enabling automated signal processing, anomaly detection, and adaptive calibration for improved diagnostic accuracy and reliability. This review presents a comprehensive overview of cutting-edge microfluidic sensor technologies for micropollutant detection, emphasizing fabrication strategies, sensing mechanisms, and their application across diverse pollutant categories. We also address current challenges, such as device robustness, scalability, and potential signal interference, while highlighting emerging solutions including biodegradable substrates, modular integration, and AI-driven interpretive frameworks. Collectively, these innovations underscore the potential of microfluidic sensors to redefine environmental diagnostics and advance sustainable pollution monitoring and management strategies.

## 1. Introduction

The increasing prevalence of micropollutants in the environment has become a pressing global issue, with far-reaching implications for ecosystems and human health [1]. Micropollutants, which include heavy metals, pesticides, pharmaceuticals, personal care products, and industrial chemicals, are often present in trace amounts, yet their cumulative effects pose severe ecological risks, such as bioaccumulation and antibiotic resistance, alongside human health threats like endocrine disruption and carcinogenicity [2]. Industrial expansion, agricultural runoff, and population growth have intensified environmental pollution, complicating monitoring efforts due to the diversity and low concentrations of these contaminants.

Conventional analytical techniques such as liquid chromatography (LC), gas chromatography (GC), and mass spectrometry (MS) remain the gold standard for micropollutant detection due to their exceptionally high sensitivity (e.g., sub-ppb detection limits) and accuracy [3]. However, these methods are labor-intensive, require expensive instrumentation, and rely on centralized laboratories, making them unsuitable for rapid on-site analysis, large-scale monitoring, or resource-limited settings. Additionally, their reliance on complex, multi-step sample preparation procedures and extensive use of hazardous organic solvents (e.g., acetonitrile) and reagents not only increases operational costs but also raises environmental concerns due to secondary pollution. As emerging contaminants (e.g., per- and polyfluoroalkyl substances [PFAS] or microplastics) and evolving regulatory thresholds amplify the complexity of environmental pollution, the limitations of conventional methods underscore the urgent need for innovative alternatives. Effective environmental monitoring now demands tools that combine sensitivity and selectivity with portability, affordability, and real-time operational capabilities [4,5]. The development of such technologies is critical to enabling timely regulatory interventions, data-driven policymaking, and the sustainable protection of public health and ecosystems.

Microfluidic technology has emerged as a revolutionary approach in analytical science, overcoming critical limitations of traditional methods such as operational inflexibility and scalability constraints [6,7,8]. At its core, microfluidics involves the precise manipulation of fluids within microscale channels (typically 10–500 μm in width), enabling the miniaturization of complex laboratory workflows (e.g., sample preparation, separation, and detection) into monolithic, portable devices [9]. These systems, often termed “lab-on-a-chip” platforms, offer transformative advantages, including ultra-low sample/reagent consumption, dramatically reduced analysis time, field-deployable portability, and seamless integration with automation or complementary technologies. 

When integrated with biosensors—devices that utilize biological recognition elements such as enzymes [10], antibodies [11,12], or aptamer systems [13,14,15]—microfluidic platforms provide highly sensitive and selective detection of target analytes [16,17]. This synergy allows for developing advanced microfluidic biosensors capable of real-time, multiplexed monitoring of complex environmental matrices. By combining the specificity of biorecognition with the precision of microfluidic control, these systems rival the analytical performance of traditional methods while addressing the critical demands of modern environmental monitoring.

One of the most significant benefits of microfluidic sensors lies in their remarkable versatility and adaptability across diverse environmental applications [18]. These platforms can be customized to detect diverse contaminants—from inorganic heavy metals to organic micropollutants—across heterogeneous environmental matrices, including aqueous systems, atmospheric particulates, and terrestrial ecosystems [19,20]. For example, they enable real-time monitoring of water quality by detecting trace levels of heavy metals or pharmaceutical residues in wastewater, as well as the surveillance of airborne pollutants and soil contaminants [21]. This multi-matrix compatibility offers a unified strategy for comprehensive environmental monitoring. Furthermore, the integration of microfluidics with advanced functional materials, such as plasmonic nanoparticles, molecularly imprinted polymers (MIPs), and two-dimensional nanomaterial, has further enhanced their analytical capabilities [22,23,24,25]. These nanomaterials contribute to signal amplification, improve target selectivity through molecular recognition or catalytic effects, and enhance operational stability by reducing biofouling in complex matrices [26]. As a result, microfluidic sensors achieve ultra-trace detection limits even under high-interference conditions.

In addition to their analytical strengths, microfluidic sensors offer substantial practical benefits for field deployment. Their compact and portable format enables rapid, on-site testing without the delays or logistical burdens associated with transporting samples to centralized laboratories [27]. This facilitates high-frequency, real-time monitoring at distributed locations, supporting prompt identification of pollution events and timely mitigation responses. Moreover, their operation requires minimal volumes of samples and reagents—often in the nano- to femtoliter range—thereby reducing material costs and aligning with green chemistry principles by minimizing waste and hazardous solvent use [28]. Collectively, these attributes position microfluidic sensors as sustainable and cost-effective alternatives to conventional analytical methods.

This review provides a comprehensive analysis of cutting-edge advancements in microfluidic-based sensors for micropollutant detection, emphasizing breakthroughs in design, fabrication, and material integration that have propelled these technologies to the forefront of environmental monitoring. Although earlier reviews have examined the utility of microfluidic platforms, our work uniquely offers a comprehensive and timely synthesis of the most recent developments in environmental microfluidic biosensing specifically for micropollutant detection over the past five years. For instance, Aryal et al. provided a broad overview of the application of microfluidic devices across various matrices, including water, air, and soil; however, their analysis did not consider advanced nanomaterial-functionalization strategies [21]. In a different approach, Mesquita et al. emphasized cost-effective fabrication techniques using paper and plastic substrates, yet they overlooked the potential for integrating plasmonic nanoparticles or graphene [29]. Meanwhile, Liu and Zhang concentrated on plasmonic biosensing within biomedical microfluidic systems, failing to address the relevant environmental matrices or the development of field-deployable device formats [30].

**Figure 1:**
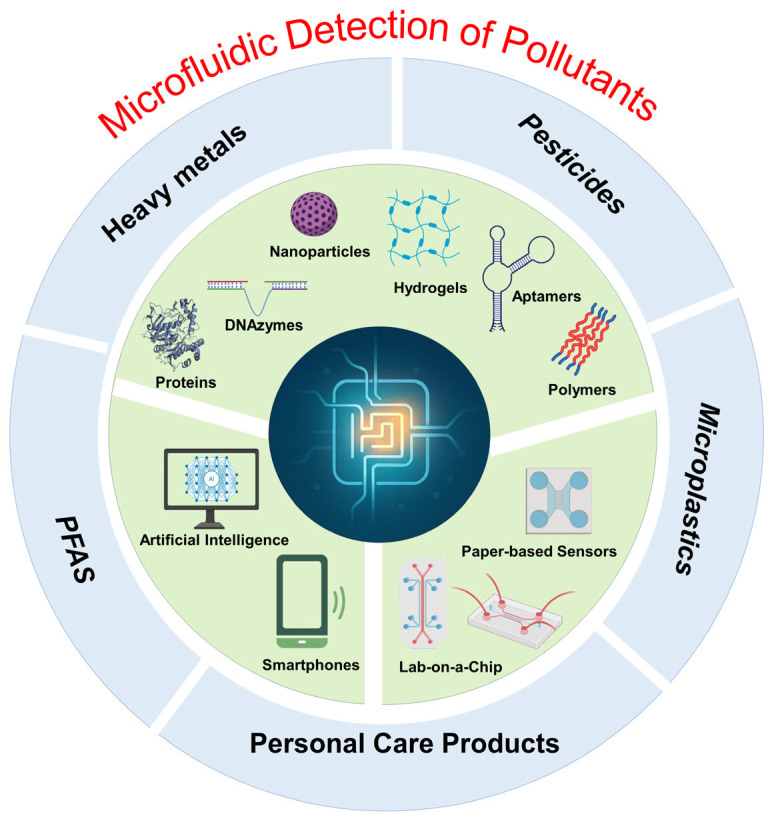
Schematic illustration of microfluidic sensors for micropollutant detection. At the core, microfluidic platforms serve as the foundational technology enabling integrated environmental sensing. Surrounding components represent key elements that enhance biosensing performance and portability, including smartphone-based interfaces, artificial intelligence (AI), and lab-on-a-chip systems, as well as functional biorecognition elements such as DNAzymes, aptamers, proteins, polymers, hydrogels, and nanoparticles. The schematic also illustrates the primary classes of targeted micropollutants: heavy metals, pesticides, per- and polyfluoroalkyl substances (PFAS), microplastics, and personal care products (PCPs).

We critically evaluate novel microfluidic platforms, including low-cost paper-based systems, high-throughput lab-on-a-chip devices, and field-deployable portable tools, while highlighting their transformative applications in detecting contaminants across water, air, and soil matrices [31,32]. A dedicated focus is placed on the strategic integration of advanced functional materials, such as plasmonic nanomaterials, graphene-based architectures, and biomimetic polymers, that enhance sensitivity, selectivity, and operational stability in complex environmental samples. Emerging innovations, such as smartphone-coupled detection systems, are also explored for their potential to democratize environmental monitoring through user-friendly interfaces, cloud-based data sharing and AI-driven real-time analysis [33,34]. As illustrated in Figure 1, the schematic consolidates these critical components—microfluidic platforms, biosensing technologies (e.g., aptamers, proteins, hydrogels, and nanomaterials), and portable digital interfaces—within an integrated framework for micropollutant detection. By synthesizing these developments, this review identifies persistent challenges (e.g., scalability, biofouling, regulatory validation) and outlines future research directions to accelerate the translation of microfluidic sensors from lab-scale prototypes to globally impactful environmental solutions.

## 2. Recent Advances in Microfluidic Sensor Fabrication and Design

### 2.1. Materials for Microfluidic Device Fabrication

The fabrication of microfluidic devices for sensor applications involves a diverse range of materials, each tailored to specific needs [35]. These materials include organic polymers such as polydimethylsiloxane (PDMS), polymethylmethacrylate (PMMA), and polyethylene terephthalate (PET), as well as inorganic substrates such as glass, silicon, ceramics, and paper [36,37,38]. Among these, PDMS is particularly popular due to its low cost, biocompatibility, and ease of prototyping. In contrast, glass and silicon offer superior chemical resistance and optical transparency, making them suitable for applications involving aggressive chemical environments or requiring precise optical detection [39]. A notable advancement in the field is the rise in paper-based microfluidic analytical devices (µPADs), which are gaining widespread attention for their affordability, portability, and environmental friendliness. Their biodegradability and ease of disposal further support their use in resource-limited settings and point-of-care diagnostics [38]. Material selection depends on application-specific factors, including chemical compatibility, optical properties, and fabrication complexity

The shift towards low-cost materials like paper and plastics, alongside advancements in affordable fabrication techniques, is broadening access to environmental monitoring [40]. Additionally, the integration of hybrid materials is emerging as a promising approach to optimize device performance [41,42]. By combining materials with complementary properties, such as enhanced chemical stability or improved detection sensitivity, microfluidic devices can better address specialized applications and detect a wider range of contaminants.

### 2.2. Microfabrication Techniques

A wide array of microfabrication techniques is employed in the development of microfluidic devices for microfluidic sensors, each presenting unique advantages and trade-offs concerning resolution, cost, scalability, and application specificity. The most commonly utilized methods include soft lithography, photolithography, 3D printing, laser ablation, and inkjet printing [43]. Soft lithography, particularly using polydimethylsiloxane (PDMS), remains a cornerstone technique for rapid prototyping and small-scale production due to its simplicity, low cost, and high biocompatibility, making it especially suitable for biological assays and early-stage microfluidic sensor development. However, PDMS suffers from limitations in scalability and durability, particularly its tendency to swell in certain solvents [44]. In contrast, 3D printing has gained significant attention for its capacity to fabricate complex and customized microfluidic architectures [45]. This additive manufacturing approach enables rapid design iteration and customization, offering greater flexibility than traditional lithographic techniques [30]. The ability to incorporate intricate internal features enhances mixing, reaction kinetics, and detection sensitivity, proving invaluable for both research and applied settings, although its resolution may still fall short of photolithography, limiting its use in high-precision applications.

Meanwhile, laser ablation is increasingly favored for producing low-cost, disposable platforms, especially in the realm of paper-based microfluidics. With the capability for precise material removal and recent innovations like two-sided patterning and acid-free fabrication, laser ablation simplifies production and promotes environmentally sustainable manufacturing, though the high initial setup and maintenance costs remain significant concerns [46,47]. Similarly, inkjet printing has emerged as a promising approach for creating low-cost, disposable microfluidic devices by enabling the direct deposition of functional materials onto various substrates, which supports flexible design and minimizes waste [48]. While ideal for rapid prototyping, inkjet printing’s lower resolution and accuracy can limit the performance of the final device [49].

Moreover, recent advancements such as picosecond and femtosecond laser processing offer exceptional precision and the ability to fabricate complex microstructures with minimal thermal damage, providing critical advantages for applications that demand high-resolution features and intricate designs [50,51]. Despite the higher costs and longer processing times, such cutting-edge methods are propelling the field toward more sophisticated and field-deployable microfluidic sensors. As microfabrication techniques continue to evolve, their strategic selection—guided by specific application needs and trade-offs—will be essential to advancing the development and deployment of next-generation microfluidic sensing platforms for environmental monitoring [52].

### 2.3. Design Strategies for Enhanced Sensitivity and Specificity

Optimizing microfluidic device design is critical to improving the sensitivity and specificity of microfluidic sensors [53]. Effective designs aim to maximize interactions between the target analyte and the biosensing element, thereby lowering detection limits. Key strategies include miniaturizing reaction chambers to concentrate analytes, increasing the surface area-to-volume ratio to enhance molecular binding, and precisely controlling fluid dynamics to facilitate efficient analyte transport to the sensor surface [54,55].

The incorporation of microfluidic components such as micropumps, microvalves, and micromixers enhances fluid handling precision and ensures uniform analyte distribution [56]. Capillary-driven systems offer an alternative approach, particularly for point-of-care or field applications, by enabling passive flow without external power sources [57]. Integrating sample preparation steps, such as on-chip extraction and preconcentration, directly into the device simplifies the analytical process and is especially advantageous for detecting trace pollutants in complex environmental matrices [58]. Furthermore, multiplexed detection capabilities enable simultaneous analysis of multiple contaminants, providing a comprehensive overview of environmental quality in a single run [59,60,61]. This approach conserves time, reagents, and operational costs, making it highly effective for monitoring diverse pollutants in water, air, and soil samples.

## 3. Application of Microfluidic-Based Sensors for Micropollutant Detection

### 3.1. Micropollutants

Micropollutants, often referred to as emerging contaminants, arise from a diverse array of anthropogenic and natural sources, reflecting the intricacies of modern industrial, domestic, and agricultural activities [62]. Among the most significant contributors is domestic and urban wastewater, which commonly contains residues of pharmaceuticals, personal care products (PPCPs), and household chemicals [62,63]. Conventional wastewater treatment plants (WWTPs), while essential for pollution control, are often inadequate in fully removing these contaminants [64,65]. As a result, treated effluents become continuous sources of micropollutants in aquatic ecosystems. Routine household items—such as detergents, cleaning agents, cosmetics, and synthetic fragrances—exacerbate this issue through regular discharge into sewage systems that ultimately reach surface waters [66].

Agriculture is another major contributor, where the extensive use of agrochemicals such as pesticides, herbicides, and fertilizers results in chemical leaching and surface runoff into nearby water bodies [67]. Additionally, veterinary pharmaceuticals—including antibiotics and hormones—enter the environment via animal waste, further increasing the micropollutant load in soil, groundwater, and surface waters [68].

Industrial effluents, particularly from chemical manufacturing, pharmaceutical production, textile processing, and mining, also play a significant role [69,70]. These waste streams often contain complex mixtures of heavy metals, solvents, dyes, and persistent organic pollutants (POPs) [71,72]. Many of which are notable for their resistance to degradation and potential to bioaccumulate, posing long-term ecological and health risks [73,74].

Improper pharmaceutical disposal, such as flushing unused medications or discarding them in landfills, further exacerbates contamination with biologically active compounds that resist degradation.

Micropollutants encompass a wide range of substances with distinct physicochemical properties and environmental behaviors [75]. Prominent categories include PPCPs—antibiotics, analgesics, antidepressants, hormones, and synthetic additives like triclosan and parabens—which are biologically active and have been shown to disrupt endocrine function in aquatic organisms [76,77,78]. Pesticides and herbicides are similarly persistent and toxic, affecting non-target species such as insects, birds, and aquatic life [79,80,81]. Heavy metals—such as mercury, lead, cadmium, and arsenic—are particularly concerning due to their non-biodegradability and potential to bioaccumulate and biomagnify [82]. POPs, including polychlorinated biphenyls (PCBs), dioxins, and flame retardants, are resistant to degradation and capable of long-range environmental transport [83]. Microplastics and nanoplastics have emerged as multipurpose contaminants: not only are they ingested by marine organisms, but they also act as carriers for other micropollutants, amplifying their ecological impact [84].

The environmental consequences of micropollutants are profound and wide-ranging. Aquatic ecosystems are particularly vulnerable, as many micropollutants enter water bodies through direct discharge or runoff [85]. For example, antidepressant exposure alters fish behavior and physiology, while antibiotics promote antibiotic-resistant bacteria, posing global public health risks [86,87]. Pesticides can lead to fish mortality and food web disruption, while nutrient overloading from fertilizers induces eutrophication and hypoxia [88,89]. Terrestrial systems also suffer; persistent pollutants degrade soil quality, disrupt microbial communities, and threaten agricultural productivity and ecosystem balance [90,91,92]. Through bioaccumulation, these substances ascend the food chain, ultimately affecting apex predators, including humans. Chronic human exposure to contaminated water, food, or air is associated with neurotoxicity (e.g., from lead and mercury), endocrine disruption (e.g., from BPA and phthalates), developmental disorders, cancers, and metabolic diseases [93,94,95]. Particularly alarming is the role of environmental antibiotic contamination in fostering antibiotic resistance [96,97,98,99,100]. Continuous, low-level antibiotic exposure in soil and water accelerates microbial adaptation, resulting in resistant strains that compromise the effectiveness of critical medical treatments [96].

In conclusion, the origin, composition, and behavior of micropollutants underscore the critical need for advanced monitoring and mitigation strategies. Their persistence, mobility, and biological activity make them formidable threats to environmental and public health. As elaborated in the following sections, microfluidic sensors present a promising avenue for real-time detection and monitoring, enabling early intervention and more effective pollution management frameworks.

### 3.2. Environmental Applications of Microfluidic Sensors

Microfluidic sensors have emerged as powerful tools for sensitive, rapid, and low-volume detection of various environmental micropollutants. Their portability and ease of use make them well-suited for on-site monitoring of contaminants such as pesticides, personal care products, heavy metals, micro/nanoplastics, and PFAS. This section reviews recent advances in these applications (Table 1).

#### 3.2.1. Agricultural Pesticides

Agricultural practices rely heavily on pesticides and herbicides to enhance crop yields; however, their extensive use leads to significant environmental contamination. Runoff from agricultural fields introduces classes of pesticides such as organophosphates, carbamates, and triazines, which pose risks to non-target species and human health. Many pesticides are persistent in the environment and capable of bioaccumulation, disrupting food chains and ecosystems.

Microfluidic platforms offer a portable and cost-effective solution for pesticide detection. A recent study developed a portable microfluidic colorimetric sensor for the rapid and precise detection of organophosphorus pesticide residues, specifically using glufosinate-ammonium as the model analyte [101]. The chip, fabricated using an LCD photo-curing system and optimized through computational fluid dynamics, featured an efficient microchannel design. It showed strong linearity (R^2^ = 0.985) between pesticide concentration and voltage, with a detection limit of 0.045 mg·L^−1^ and a detection time of just 60 s. Compared to conventional chromatographic methods, this platform offers a faster, lower-cost, and more field-deployable alternative. Another study by Yang et al. reported the development of an enzyme-free ratiometric fluorescent detection system integrated with a hinge-like dual-channel 3D microfluidic paper-based analytical device (3D μPAD) for the simultaneous on-site detection of carbaryl and glyphosate [102]. The system relied on distinct fluorescence emissions and inner-filter effects between reaction products and carbon dots to enable visual, ratiometric detection with clear color changes. Each microchannel responded independently without signal crosstalk, allowing dual-analyte detection in a single platform. The method achieved low LODs of 1.11 μM for carbaryl and 0.63 μM for glyphosate, with high accuracy and precision demonstrated in spiked food and herbal samples. The platform’s reagent efficiency, portability, and dual-analyte capacity position it as a promising tool for real-world pesticide surveillance.

Organophosphorus pesticides, such as malathion, are widely used insecticides that pose environmental and health risks due to their persistence and toxicity. Demonstrating a novel analytical approach, a recent study developed the first dual-mode microfluidic biosensor integrating a target-triggered DNA hydrogel for simultaneous colorimetric and electrochemical detection of malathion [103]. The sensor employed aptamer-functionalized DNA hydrogels encapsulating gold nanoparticles and ferrocene to achieve dual signal outputs. The colorimetric mode enabled rapid visual detection suitable for portable on-site analysis, with a smartphone-assisted readout yielding a detection limit of 56 nM. The electrochemical mode provided enhanced sensitivity with a wide linear range (0.01–3000 μM) and a low detection limit of 5 nM. These two detection modes validated each other, improving reliability and reducing false results. The hydrogel’s three-dimensional network supported aptamer stability and minimized environmental interferences. Integrated into a microfluidic chip, the system offered portability, user-friendliness, and reduced procedural complexity without compromising sensitivity or accuracy. This dual-mode platform represents the first reported integration of colorimetric and electrochemical outputs in a single microfluidic biosensor for organophosphorus pesticide detection, marking a significant advancement in multifunctional environmental sensing technologies.

Recent advancements in integrating microfluidic systems with cutting-edge detection techniques have enabled powerful solutions for the rapid and sensitive analysis of environmental contaminants. In a recent study, a highly integrated microfluidic glass chip was developed to detect phenylurea herbicides (PHs) by synchronously combining liquid chromatography (LC), electrochemical detection (ECD), and surface-enhanced Raman spectroscopy (SERS) within a single platform [104]. This system achieved complete separation of three PHs with an impressive theoretical plate number of 342,525 plates/m and demonstrated electrochemical detection limits ranging from 9.9 μM to 138.8 μM. The integration with SERS significantly enhanced detection capabilities, as this technique amplifies Raman signals of molecules adsorbed on metallic surfaces, allowing for ultra-sensitive detection and molecular specificity based on unique vibrational fingerprints. Such enhancement enabled single-molecule-level detection, a notable advantage for distinguishing structurally similar pesticide residues. In real environmental samples, including water and soil extracts, the platform demonstrated strong matrix tolerance and achieved recoveries of up to 102.99%, confirming its analytical reliability. This multi-modal microfluidic platform shows considerable promise for environmental safety monitoring, food residue analysis, and biomedical diagnostics, especially when analyzing complex sample matrices with potential interferences.

**Table 1:** Emerging Microfluidic-Based Detection Technologies for Environmental Pollutants.

Micropollutant Type	Chip Materials	Detection Method	Target Micropollutant	LOD	Portability	References
Agricultural Pesticides	LCD-fabricated microfluidic mixer chip	Colorimetric detection with a photodetector	Organophosphorus pesticide residues	0.045 mg·L^−1^	+/(battery-powered)	[101]
	3D microfluidic paper analytical device (3D μPAD)	Enzyme-free ratiometric fluorescence	Carbaryl, Glyphosate	1.11 μM (Carbaryl), 0.63 μM (Glyphosate)	+/(Smartphone-assisted portable system with custom app)	[102]
	Microfluidic chip, aptamer-functionalized DNA hydrogel and AuNPs	Colorimetric and electrochemical	Malathion	56 nM (colorimetric), 5 nM (electrochemical)	+/(Smartphone-based colorimetric sensor)	[103]
	Microfluidic glass chip	LC-ECD-SERS integrated detection	Phenylurea Herbicides	9.9 μM–138.8 μM	–/(Lab-based, complex instrumentation)	[104]
	3D paper-based chip with metal–organic frameworks	Dual-mode: fluorescence and colorimetric detection	CPF	0.028 ng/mL (fluorescence), 0.043 ng/mL (colorimetric)	+/(Field-deployable, portable readout)	[105]
	μ-PAD, polystyrene-AuNP microparticles	Smartphone-integrated colorimetric aptasensor	Imidacloprid, Carbendazim	3.12/1.56 ppm	+/(Smartphone-based portable system)	[106]
	Polyimide (PI) film with LIG-Au electrodes and PDMS channels	Electrochemical detection via smartphone	Methyl Parathion	0.000646 μM	+/(Capillary-driven, on-site detection)	[107]
Personal Care Products	Paper-based μPAD with AgNPs/MWCNT ink; ZnO-modified zones	Dual: Electrochemical & LDI-MS detection via capillary action	BPA	0.35 μM (electrochemical), 0.01 pM (LDI-MS)	−/(Requires LDI-MS; not field-portable)	[108]
	Agarose membrane with natural deep eutectic solvents	Green microfluidic-based liquid-phase microextraction (LPME)	Parabens	0.011 μg/mL	−/(Lab-bound setup, no integrated readout)	[109]
	2D Ni-BDC-NH_2_ MOF grown via LbL-LPE on Si/SiO_2_ with microelectrode arrays in a microfluidic chip	Electrochemical impedance spectroscopy (EIS)	DiBP	1–20 μg/mL (range)	−/(Bench-scale fabrication, not field-portable)	[110]
	Screen-printed paper device with buckypaper working electrode	Electrochemical detection via potentiostat	Phthalates	12.64 ppm	+/(Screen-printed, low-voltage, field-tested)	[111]
Heavy Metals	Portable microfluidic chip with DNAzyme and magnetic/polystyrene microparticles	Visual thermometer-like readout via DNAzyme-based CHA	Cd^2+^	11.3 nM	+/(Compact chip with visual readout for on-site detection)	[112]
	PMMA and microfluidic chip and electronics	Chemiluminescence	Cd^2+^	0.207 µg/L	+/(Portable workstation with remote control and on-site applicability)	[113]
	Microfluidic chip, graphene oxide (GO), aptamers labeled with FAM/HEX dyes	FRET-based fluorescence quenching by GO-aptamer interactions	Hg^2+^ and Pb^2+^	0.70 ppb (Hg^2+^), 0.53 ppb (Pb^2+^)	+/(Compact PDMS chip enabling on-site fluorescence sensing)	[114]
	3D-printed microfluidic device, silver nanoparticles (AgNPs@BS), leucomalachite green dye	Naked-eye colorimetric detection (color change)	Hg^2+^ and As^3+^	0.089 mg/L (Hg^2+^), 0.042 mg/L (As^3+^)	+/(3D-printed portable chip with smartphone quantification)	[115]
	Hydrophobin chimera on polystyrene multiwell plates	Fluorescence decrease coupled with machine learning analysis	Hg^2+^	0.3 nM (seawater), 0.4 nM (tap water)	+/(Smartphone-assisted portable biosensor with ML support)	[116]
	PDMS microchannel and organic probes	Fluorescence	Hg^2+^, Pb^2+^, Cr^3+^, Cu^2+^	0.89 nM (Hg^2+^), 9.60 nM (Pb^2+^), 5.45 nM (Cr^3+^), 1.77 nM (Cu^2+^)	+/(Smartphone-based detection system)	[117]
Microplastics & Nanoplastics	PDMS microchannel with copper microwire electrodes	Electrical resistance measurement under DC electrophoretic force	Polystyrene microplastics (1–10 μm)	Not specified	−/(Lab-based setup)	[118]
	Microfluidic channel integrated with microwave electric inductive-capacitive resonator	Microwave sensor detecting S11 resonance frequency shift	Microplastics	Not specified	+/(Compact, suitable for real-time use)	[119]
	Fiber-embedded optofluidic chip	180° laser-backscattered signal quantification	PS, PE, PET, PP, PMMA	0.23 μg/mL (PS)	+/(Compact, no reagents, field applicable)	[120]
	Droplet-based microfluidic system (PDMS)	ML-assisted real-time droplet image analysis	Polystyrene MPs (3–50 μm)	Not specified	+/(AI-assisted, portable, field validated)	[121]
	Agarose-based microfluidic chip with micropost arrays	ML-assisted Raman spectroscopy	Polystyrene NPs (100 nm)	6.25 µg/mL	+/(Scalable, AI-integrated, seawater-compatible)	[122]
PFAS	Janus droplet-based microfluidic chip	Real-time optical sensing, PCA, and Random Forest analysis	Four PFAS compounds	Not specified	+/(ML)	[123]
	MOF (Cr-MIL-101) + Interdigitated microelectrodes (IDμE)	Electrochemical impedance spectroscopy	PFOS	0.5 ng/L	–/(Lab-based setup; lacks field integration)	[124]

Abbreviations: LOD, limit of detection; LCD, liquid crystal display; CPF, chlorpyrifos; PDMS, polydimethylsiloxane; AuNP, gold nanoparticle; AgNP, silver nanoparticle; MWCNT, multiwalled carbon nanotube; LDI-MS, laser desorption ionization mass spectrometry; MOF, metal–organic framework; LIG, laser-induced graphene; GO, graphene oxide; FRET, fluorescence resonance energy transfer; CHA, catalytic hairpin assembly; EIS, electrochemical impedance spectroscopy; 3D μPAD, three-dimensional microfluidic paper-based analytical device; LC-ECD-SERS, liquid chromatography–electrochemical detection–surface enhanced Raman spectroscopy; PCA, principal component analysis; ML, machine learning; BPA, bisphenol A; DiBP, diisobutyl phthalate; PMMA, polymethyl methacrylate; Cd^2+^, cadmium ion; Hg^2+^, mercury ion; As^3+^, arsenic ion; Cr^3+^, chromium ion; Cu^2+^, copper ion; FAM, fluorescein amidite; HEX, hexachlorofluorescein; PS, polystyrene; PE, polyethylene; PET, polyethylene terephthalate; PP, polypropylene; PFOS, perfluorooctanesulfonic acid; PFAS; per- and polyfluoroalkyl substances.

**Figure 2:**
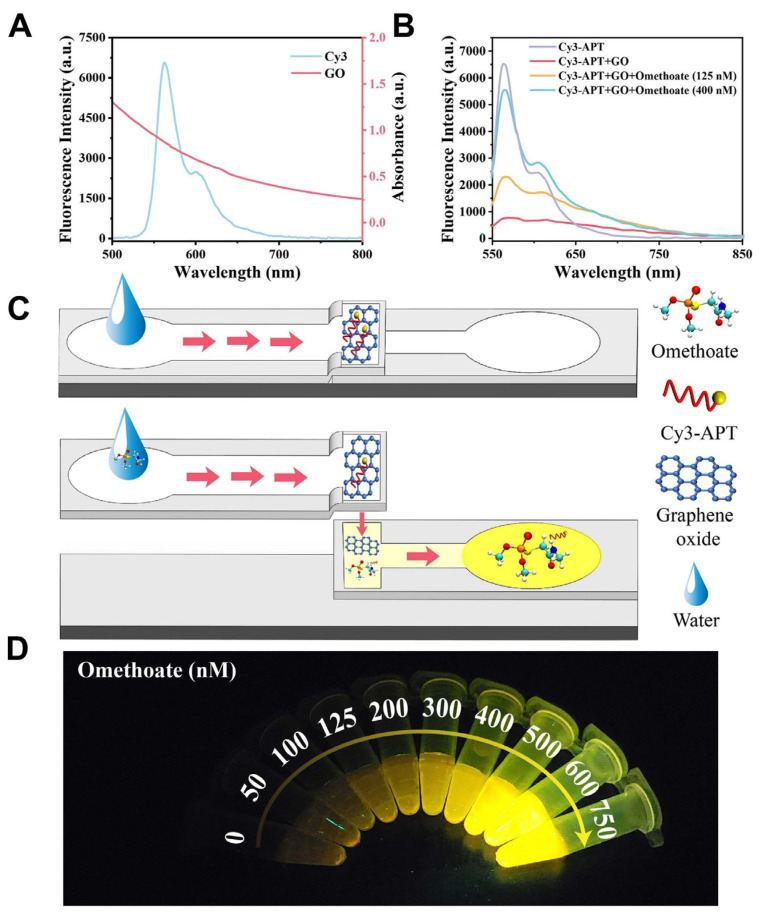
A paper-based microfluidic platform for omethoate detection. (**A**) Fluorescence and UV–Vis spectra of Cy3 and graphene oxide (GO). (**B**) Fluorescence response of the Cy3-labeled aptamer alone, with GO, and with GO plus omethoate (125 and 400 nM). (**C**) Schematic of the microfluidic chip in the absence and presence of omethoate. (**D**) Fluorescence images showing increased signal intensity with rising omethoate concentration. Reproduced with permission from [125], Copyright 2024 Elsevier Ltd.

Optical and electrochemical microfluidic sensors showed great promise for the sensitive detection of pesticides such as chlorpyrifos and omethoate. One study developed a three-dimensional folded paper-based microfluidic analytical device (3D-μPAD) using multifunctional metal–organic frameworks with peroxidase-like activity and upconversion nanomaterials to enable fluorescence-colorimetric dual-mode detection of chlorpyrifos [105]. The device exhibited strong linearity and low detection limits—0.028 ng/mL for fluorescence and 0.043 ng/mL for colorimetric mode—with performance comparable to high-performance liquid chromatography (HPLC) in spiked real samples. The dual-mode capability enhanced detection reliability and allowed for flexible signal readout under field conditions. Another study introduced a microfluidic paper strip coupled with a smartphone-based platform for omethoate detection, utilizing a Cy3-labeled aptamer quenched by graphene oxide (Figure 2) [125]. Upon omethoate binding, fluorescence was restored and analyzed using a custom smartphone app integrated with a pretrained convolutional neural network. The system demonstrated a high correlation (R^2^ = 0.9964), achieved a low LOD of 0.16 nM, and delivered on-site results in under 10 min. As illustrated in Figure 2, the detection mechanism is based on fluorescence resonance energy transfer (FRET), where Cy3 acts as the donor and graphene oxide as the quencher. Omethoate binding causes aptamer detachment from GO, restoring fluorescence. This response was captured using a two-layer microfluidic paper device, producing a strong linear fluorescence signal across 0–600 nM. These studies underscore the potential of microfluidic paper-based analytical devices (μPADs), enhanced with nanomaterials and intelligent smartphone platforms, as rapid, portable, and cost-effective tools for pesticide residue monitoring in real-world applications.

**Figure 3:**
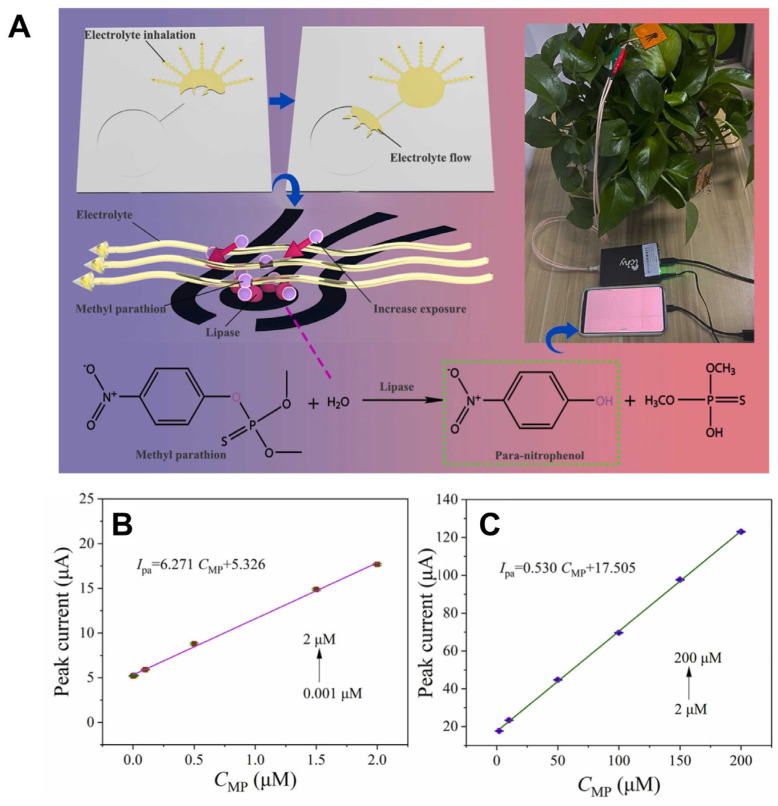
A self-adsorbing electrolyte-enabled microfluidic sensor for methyl parathion (MP) detection on plant surfaces. (**A**) Schematic of detection principle and on-leaf application. (**B**,**C**) Calibration plots for MP in the low (0.001–2 μM) and high (2–200 μM) concentration ranges. Reproduced with permission from [107], Copyright 2024 Elsevier B.V.

Recent advances in microfluidic and wearable sensors have enhanced pesticide detection in agriculture and food safety. A laser-induced graphene-gold (LIG-Au) electrode-based microfluidic sensor was designed for the in situ detection of methyl parathion (MP) on plant surfaces (Figure 3) [107]. This sensor utilized capillary action to drive electrolyte flow, enabling rapid pre-enrichment and electrochemical detection with a sensitivity range from 0.001 μM to 200 μM and a detection limit of 0.000646 μM. Similarly, a low-cost paper-based electrochemical microfluidic device (EμPAD) was created using a metal–organic framework (MOF) for immobilizing acetylcholinesterase (AChE), allowing for ultrasensitive detection of chlorpyrifos (CPF) with a limit of detection (LOD) of 3 ng/L [126]. The sensor, integrated with portable electronics and artificial intelligence, showed excellent performance in real food samples. Additionally, a sustainable, flexible sensor was

Developed using cellulose acetate (CA) as a substrate, coupled with screen-printed electrodes to detect carbendazim and paraquat in agricultural and water samples [127]. This non-enzymatic sensor demonstrated high sensitivity, stability, and selectivity, with a detection limit of 54.9 nM and 19.8 nM for carbendazim and paraquat, respectively. The use of biodegradable and biocompatible materials, such as CA, makes this technology environmentally friendly while providing reliable, decentralized pesticide monitoring in precision agriculture. These advances underscore the potential of microfluidic and printed biosensors for on-site, rapid, and cost-effective pesticide detection, offering promising solutions for food safety and environmental protection.

In response to the growing demand for comprehensive and efficient pesticide monitoring, recent advancements in microfluidic sensing have increasingly emphasized the development of multiplexed and portable platforms capable of simultaneously detecting and distinguishing multiple pesticide residues with high specificity and throughput. Innovative strategies—including spatially resolved detection zones, encoded aptamer arrays, and advanced signal amplification methods—have enabled precise analyte differentiation in complex food and environmental matrices. For instance, Cui et al. demonstrated a bio-barcode immunoassay integrated with droplet digital PCR (ddPCR) that achieved simultaneous quantification of triazophos, parathion, and chlorpyrifos in fruits and vegetables. By utilizing gold and magnetic nanoparticles functionalized with distinct antibodies and primers, the system achieved high sensitivity, reporting detection limits of 0.22, 0.45, and 4.49 ng/mL for the three respective pesticides [128]. These and other emerging platforms—including eco-friendly paper-based μPADs and plant-wearable electrochemical sensors (e.g., graphene-based flexible patches for in situ pesticide analysis—underscore the transformative potential of microfluidic technologies [129]. By combining high sensitivity, portability, and real-time capabilities, microfluidic and wearable sensors are poised to revolutionize pesticide detection across agriculture and environmental monitoring. Ultimately, these scalable, low-cost, and field-deployable tools offer promising solutions to enhance food safety, support precision agriculture, and promote sustainable pesticide management worldwide.

#### 3.2.2. Personal Care Products (PCPs)

Personal care products (PCPs), such as cosmetics, shampoos, detergents, and synthetic fragrances, are major contributors to micropollutant loads in domestic wastewater [130]. Active ingredients including triclosan, parabens, phthalates, and synthetic musks are environmentally persistent and have been linked to endocrine disruption in aquatic organisms [131,132]. Due to their widespread use and chemical stability, residues from PCPs frequently bypass conventional wastewater treatment processes and are subsequently discharged into surface waters [133,134].

**Figure 4:**
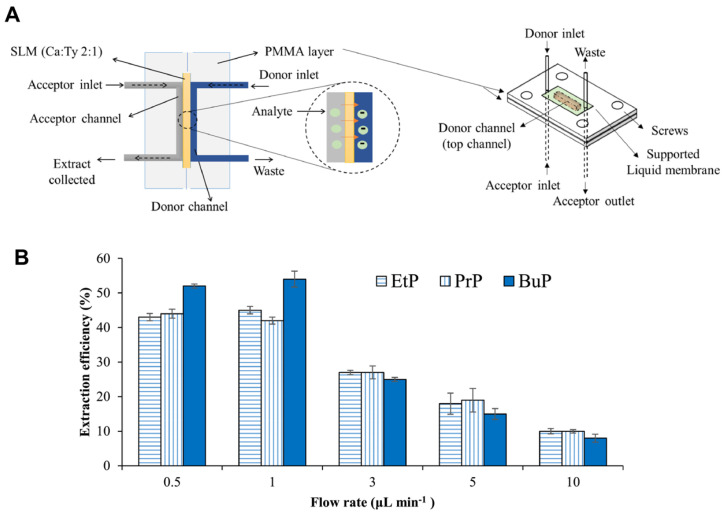
(**A**) Schematic representation of a microfluidic device for detecting parabens in human urine samples. (**B**) Extraction efficiency plotted against sample flow rate under conditions using a supported liquid membrane composed of camphor and thymol in a 2 to 1 ratio, sample pH 4, acceptor pH 12, and acceptor flow rate of 1 µL/min. Reproduced with permission from [109], Copyright 2022 Elsevier B.V.

Microfluidic technologies have emerged as powerful platforms for the sensitive, sustainable, and miniaturized detection of micropollutants derived from personal care products (PCPs), including common chemicals such as triclosan, bisphenol A (BPA), and parabens. These substances are frequently detected in water systems and are known for their endocrine-disrupting effects and environmental persistence. For triclosan detection, a low-cost, disposable paper-based electrochemical sensor was developed using a composite of graphite lubricant and silicone sealant screen-printed onto sulfite paper [135]. While not strictly a microfluidic device, its miniaturized format and suitability for on-site testing share key microfluidic characteristics. The sensor demonstrated a low detection limit of 0.05 µmol/L and high recovery rates ranging from 96% to 118% in real-world samples such as tap water, toothpaste, and mouthwash. For bisphenol A (BPA), a three-dimensional microfluidic paper-based analytical device (3D-μPAD) was fabricated using a digital plotter to enable capillary-driven flow without external pumps [108]. The platform combined electrochemical detection and laser desorption ionization mass spectrometry (LDI-MS), with printed electrodes composed of silver nanoparticles and multiwall carbon nanotube (MWCNT) ink modified by ZnO for enhanced sensitivity. The μPAD achieved detection limits of 0.35 μM (electrochemical) and 0.01 pM (LDI-MS) using just 10 μL of sample, demonstrating high precision for trace BPA detection across a broad dynamic range. A third approach targeted parabens using a green microfluidic extraction system. The device utilized natural deep eutectic solvents (camphor: thymol, 2:1) immobilized in biodegradable agarose membranes, allowing environmentally friendly extraction from urine samples under optimized low flow conditions (1 μL/min) (Figure 4) [109]. This system achieved a detection limit of 0.011 μg/mL, with recovery rates of 86–100% and relative standard deviations (RSDs) below 7%, while avoiding toxic solvents and offering membrane reusability. Collectively, these platforms highlight the versatility and sustainability of microfluidic and paper-based devices for detecting PCP-derived micropollutants across diverse matrices. Their integration with advanced materials and dual-mode detection technologies underscores their potential for field-deployable, eco-conscious monitoring of emerging contaminants.

Microfluidic approaches for detecting PCPs have also been extended to phthalates, synthetic chemicals widely used as plasticizers in packaging, cosmetics, and consumer products. Due to their endocrine-disrupting properties and environmental persistence, phthalates such as diisobutyl phthalate (DiBP) are considered priority micropollutants with growing regulatory concern. In one strategy, programmable layer-by-layer liquid-phase epitaxy (LPE) was used to fabricate two-dimensional nickel-based metal–organic frameworks (Ni-MOFs) directly on microfluidic chips integrated with metal microelectrode arrays (MEAs) [110]. This method enabled the scalable and uniform growth of crystalline sensor arrays with thicknesses ranging from 2 to 25 nm. The resulting sensor system was applied for electrochemical impedance spectroscopy (EIS)-based detection of DiBP, with a quantifiable concentration range of 1–20 μg/m. A second approach involved a disposable, screen-printed paper-based electrochemical sensor integrated with buckypaper, operating at −0.25 V [111]. The platform achieved a linear detection range of 70–15 ppm and a limit of detection (LOD) of 12.64 ppm. Field validation using real water samples demonstrated high recovery rates and good repeatability, supporting its practical utility. Together, these microfluidic systems represent effective, scalable, and sustainable tools for phthalate monitoring in environmental and food safety applications, reinforcing the broader potential of microfluidic platforms for PCP-related pollutant detection.

#### 3.2.3. Heavy Metals and Inorganic Contaminants

Heavy metals such as mercury (Hg), lead (Pb), cadmium (Cd), and arsenic (As) are among the most hazardous micropollutants [136]. Their toxicity stems from their non-biodegradability and tendency to bioaccumulate in living organisms [137]. These metals primarily originate from industrial effluents, mining operations, and improper waste disposal. As a result, they pose serious threats to both aquatic and terrestrial ecosystems. Chronic exposure in humans has been linked to neurotoxicity, developmental disorders, and cancer.

**Figure 5:**
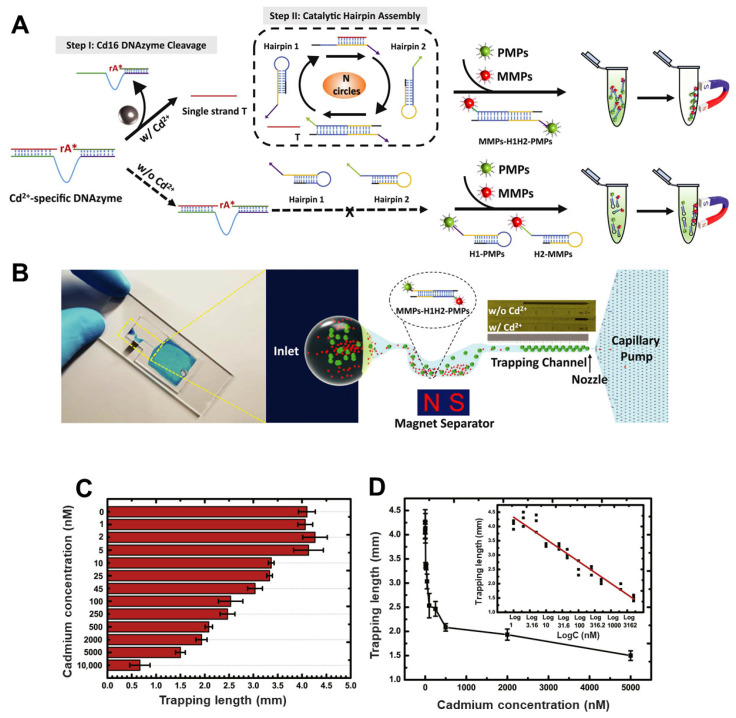
Visual quantification of cadmium ions using a microfluidic device based on DNAzyme-mediated signal amplification. (**A**) Schematic representation of the detection mechanism: Cd^2+^ ions cleave the Cd16 DNAzyme at the cleavage site (rA*), releasing a trigger strand (T) that initiates catalytic hairpin assembly (CHA) between H1 and H2 hairpins. This leads to the formation of H1–H2 complexes that link magnetic microparticles (MMPs) to polystyrene microparticles (PMPs) through the recycling of T. (**B**) Design of the microfluidic chip used for detection: a magnetic separator removes MMPs and MMP–PMP conjugates, while unbound PMPs are trapped at the nozzle, forming a visual signal in the trapping channel. (**C**) Graph showing the measured length of PMP accumulation. (**D**) Relationship between PMP accumulation length and Cd^2+^ concentration (mean ± maximum deviation, *n* = 3); inset: linear regression analysis of PMP accumulation versus the logarithm of Cd^2+^ concentration. Reproduced with permission from [112], Copyright 2021 Elsevier B.V.

Microfluidic platforms are increasingly proving their value in the sensitive, portable, and cost-effective detection of toxic heavy metals such as cadmium (Cd^2+^), particularly for on-site environmental monitoring. Three innovative microfluidic devices exemplify this advancement. The first was a portable microfluidic chip designed for real-time, visual detection of Cd^2+^ in drinking water, addressing the limitations of conventional, bulky spectrometric techniques. This device combined a Cd^2+^-specific DNAzyme (Cd16) with catalytic hairpin assembly (CHA) amplification to initiate a reaction cascade that formed H1H2 complexes (Figure 5) [112]. These complexes linked magnetic microparticles (MMPs) and polystyrene microparticles (PMPs) into a sandwich structure, which was visually quantified through a thermometer-like display on the microfluidic chip—where the accumulation of unbound PMPs inversely correlated with Cd^2+^ concentration. This system achieved a detection limit of 11.3 nM, well below the EPA safety threshold, with over 200-fold selectivity against other metal ions, strong resistance to typical water interferents, and effective performance in unprocessed tap water. Another paper-based microfluidic analytical device (μPAD) was also developed. It used ion imprinted polymers (IIPs) grafted onto paper for the selective separation of Cd^2+^ [138]. This device integrated a screen-printed carbon electrode modified with reduced graphene oxide (rGO/pSPCE) for electrochemical sensing and achieved a very low detection limit of 0.05 ng/mL, with excellent recovery rates (96.5–114.2%) in real water samples, comparable to ICP-MS analysis. Most recently, a remote-controlled portable workstation (RCPW) incorporating a microfluidic chip was introduced for real-time, point-of-care detection of Cd^2+^ in food samples [113]. This platform utilized a Luminol–KMnO_4_ chemiluminescence (CL) system, in which Cd^2+^ markedly enhanced the CL signal, enabling sensitive detection over a range of 0–0.18 mg/L with a limit of detection as low as 0.207 µg/L. The system integrated a cascade micromixer to improve reagent interaction and included a remote-control module for automated analysis, thereby eliminating the need for centralized laboratory processing. It demonstrated strong selectivity, matrix resistance, and recoveries of 87–106%, showing excellent agreement with ICP-MS. Collectively, these platforms underscored the growing potential of microfluidic systems in delivering robust, selective, and field-deployable solutions for cadmium detection in both environmental and food safety applications.

A microfluidic paper-based analytical device (μPAD) was developed for on-site detection of lead ions (Pb^2+^) in urine [139]. It combined both protein isolation and electrochemical sensing. The device was made by printing microchannel patterns onto filter paper using a standard office laser printer. The paper was then modified with a protein precipitant to allow efficient sample preparation. A detachable three-electrode system was included for electrochemical analysis. This made the device both portable and cost-effective. Key parameters such as deposition potential, deposition time, and channel size were optimized for better performance. The μPAD worked effectively with protein concentrations up to 300 mg/L, which is suitable for real urine samples. It provided a linear detection range of 10–500 μg/L for Pb^2+^ and a low detection limit of 9 μg/L. Results from urine samples matched closely with those from atomic absorption spectrometry (AAS). This confirms the device’s accuracy and reliability. Overall, this study shows the strong potential of microfluidics for low-cost, portable detection of heavy metals in biological samples. It opens the way for applications in health monitoring and environmental screening.

**Figure 6:**
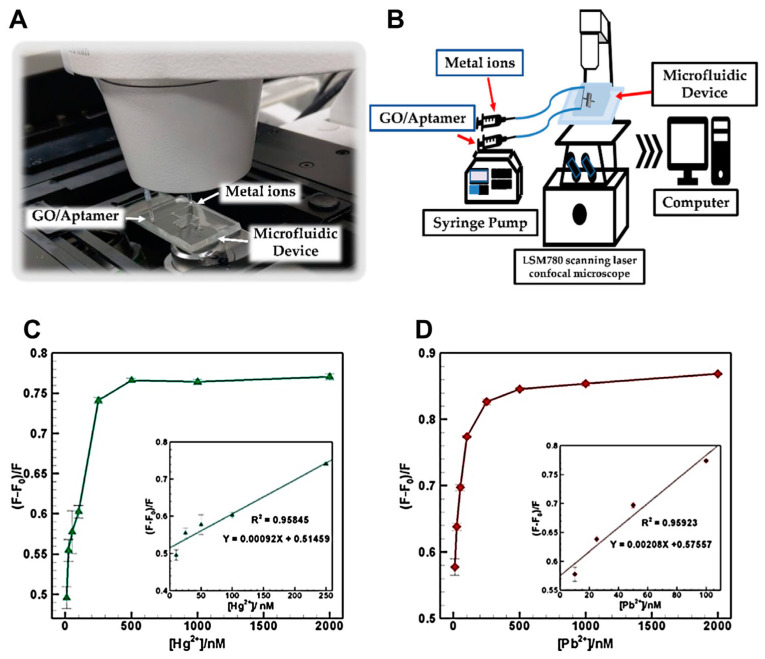
Microfluidic aptasensor for Hg^2+^ and Pb^2+^ detection in water. (**A**) Detection setup. (**B**) Schematic of system integration. (**C**) Fluorescence recovery for Hg^2+^ (10–2000 nM); inset: calibration curve (10–250 nM). (**D**) Fluorescence recovery for Pb^2+^ (10–2000 nM); inset: calibration curve (10–100 nM). Reproduced with permission from [114], Copyright 2020 MDPI (CC BY 4.0).

A novel microfluidic-based sensor was developed for the simultaneous detection of mercury (Hg^2+^) and lead (Pb^2+^) ions using a fluorescence resonance energy transfer (FRET) approach (Figure 6) [114]. The system integrated graphene oxide (GO) as a quencher and fluorescent dye-labeled aptamers (FAM for Hg^2+^ and HEX for Pb^2+^) within a PDMS microfluidic device. Upon interaction with their target ions, fluorescence was restored, enabling quantification based on intensity change. The device demonstrated excellent sensitivity with detection limits of 0.70 ppb for Hg^2+^ and 0.53 ppb for Pb^2+^, both below WHO permissible levels for drinking water. It also exhibited high selectivity against other metal ions and maintained linear detection ranges of 2.0–50 ppb for Hg^2+^ and 2.1–20.7 ppb for Pb^2+^. These findings confirmws the device’s potential as a portable, selective, and sensitive microfluidic platform for on-site heavy metal monitoring in environmental samples.

Al-aqbi et al. introduced a low-cost, portable, and environmentally friendly 3D-printed microfluidic device for the on-site colorimetric detection of mercury (Hg^2+^) and arsenic (As^3+^) in Tigris River water samples [115]. The device featured two V-shaped microchannels, enabling dual detection functions. For mercury sensing, it employed silver nanoparticles (AgNPs) stabilized with Boswellia sacra extract; the presence of Hg^2+^ caused AgNP aggregation, resulting in a visible color change from brown to colorless. For arsenic detection, the device used a leucomalachite green (LMG) dye system, where As^3+^ induced a chemical transformation that produced a green malachite green dye. The colorimetric changes could be observed with the naked eye and were further quantifiable using smartphone imaging. The device achieved detection limits of 0.089 mg/L for Hg^2+^ and 0.042 mg/L for As^3+^, with quantification levels suitable for environmental monitoring. Comparison with ICP-MS analysis showed a 97% correlation, confirming the accuracy, sensitivity, and practical utility of the device for real-time water quality assessment.

A novel microfluidic electrochemical platform was developed for rapid, on-site detection and speciation of inorganic arsenic (iAs), integrating dual microfluidic channels with gold nanoparticle-modified screen-printed graphene electrodes (AuNP/SPGE) [140]. The device simultaneously quantified As(III) and total iAs within 9 min by using thioglycolic acid for on-chip reduction in As(V), enabling speciation by subtraction. Wireless control via a smartphone-connected Bluetooth potentiostat allowed automated, field-friendly operation. The platform demonstrated good sensitivity with detection limits of 3.7 ng/mL for As(III) and 17 ng/mL for total iAs, and its performance was consistent with standard HPLC-ICP-MS analysis of food samples. Although the detection limit for total iAs exceeded the WHO guideline, the system offered a unique, cost-effective, and portable solution for rapid inorganic arsenic speciation, highlighting the potential of microfluidic technologies in environmental and food safety monitoring.

Expanding on the use of microfluidic systems for heavy metal detection, another recent study introduced a portable fluorescence-based sensor array tailored for the rapid, on-site, and simultaneous quantification of multiple toxic metal ions [117]. By integrating selective organic fluorescence probes into a patterned microchannel network, the platform enables capillary-driven sample flow without the need for pretreatment. Upon interaction with target ions, the system exhibits distinct fluorescence “light-up” responses within 3 s, producing visible color changes from colorless to green (Pb^2+^), red (Cr^3+^), and blue (Cu^2+^). Quantitative analysis reveals limits of detection as low as 9.60 nM for Pb^2+^ (color shift: colorless to green, emission at 485 nm, R^2^ = 0.9913), 5.45 nM for Cr^3+^ (colorless to red, emission at 610 nm, R^2^ = 0.9899), and 1.77 nM for Cu^2+^ (colorless to blue, emission at 450 nm, R^2^ = 0.9889), calculated using the standard 3σ/k method. The entire detection process is completed in under one minute, with performance validated by high recoveries (95.20–120%) and low RSDs (0.89–6.74%). Moreover, coupling the array with a handheld intelligent terminal enables RGB data capture and quantification, demonstrating excellent potential for real-time, user-friendly monitoring of heavy metals in complex environmental samples.

#### 3.2.4. Microplastics and Nanoplastics

Microplastics (MPs) and nanoplastics (NPs) are emerging classes of environmental micropollutants, primarily originating from the degradation of larger plastic items, synthetic textiles, and personal care products (PCPs). These particles not only pose physical hazards to aquatic organisms but also serve as carriers for secondary pollutants such as heavy metals and hydrophobic organic contaminants, thereby amplifying their ecological and toxicological impact. Their ubiquity in environmental matrices—including water, soil, and air—combined with their diverse sizes, shapes, and compositions, presents a major analytical challenge for accurate detection and quantification.

Microfluidic technologies are increasingly being applied to detect and characterize microplastics in environmental samples. These plastics, especially those in the 1–10 μm range, are known to pose ecological and health risks due to their persistence and their tendency to accumulate toxic compounds. However, their detection is complicated by the presence of similarly sized natural particulates such as plankton, minerals, and organic matter. In one approach, a simple DC electrophoresis-based microfluidic sensor was developed using copper microwire electrodes embedded in a wide PDMS channel to electrically detect polystyrene MPs [118]. As MPs accumulated electrophoretically near the positive electrode, their presence was quantified via changes in electrical resistance, which showed a strong correlation with MP concentration. This platform provided a rapid, low-cost, and scalable alternative to traditional optical methods, with the added advantage of potential integration into handheld, on-site detection devices. A second platform utilized a PDMS microfluidic chip integrating a trap module with SU-8 grooves and a mixer module for in situ fluorescent labeling of polyethylene (PE), polypropylene (PP), and polystyrene (PS) MPs [141]. The system achieved up to 69% trapping efficiency and enabled efficient Nile Red staining and PS-specific detection using peptide-conjugated gold nanoparticles. Its compact format and reagent-saving design make it well-suited for lab-on-a-chip applications targeting microplastic identification and quantification. A third study developed a microfluidic microwave detection sensor optimized around a 4.7 GHz electric inductive-capacitive resonator [119]. The device demonstrated high sensitivity through resonance frequency shifts (~10 MHz per 1% MP concentration change), with excellent linearity (R^2^ ≈ 0.999) confirmed by the Maxwell-Garnett model. This sensor offered high sensitivity and rapid operation, making it an attractive tool for real-time monitoring. Despite these advances, selectivity remains a challenge due to interference from natural particulates. Encouragingly, dual-frequency impedance cytometry coupled with machine learning has successfully differentiated MPs from phytoplankton in seawater-like media [142], and microwave cytometry has distinguished between polystyrene and polyethylene MPs [143]. These developments suggest that combining advanced microfluidic electrical detection with AI-enhanced classification offers a promising path toward more reliable microplastic analysis in complex environmental samples.

**Figure 7:**
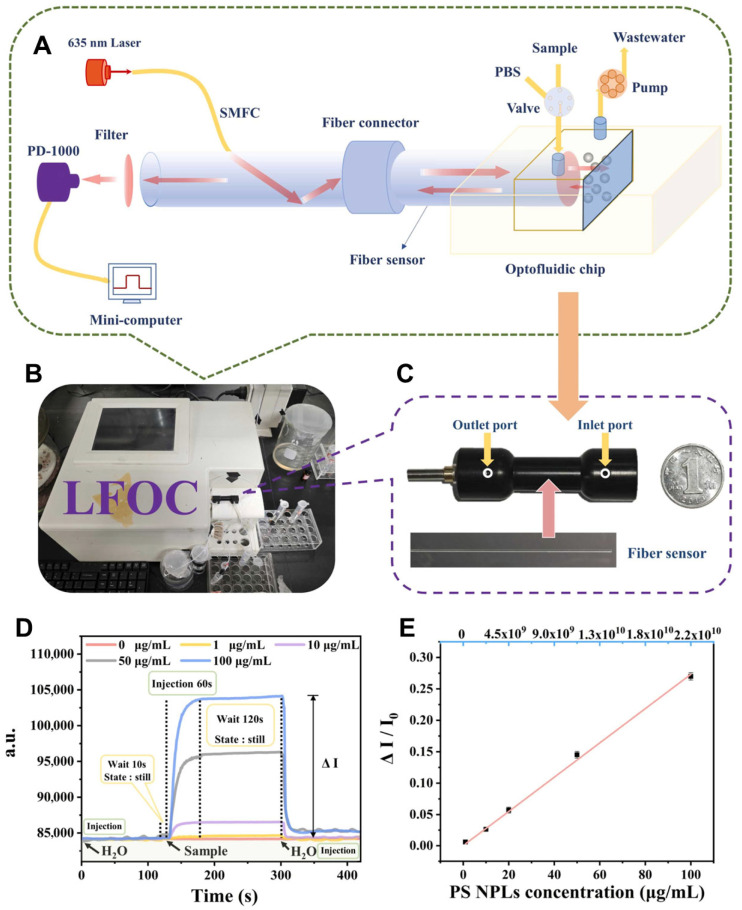
Laser-backscattered fiber-embedded optofluidic chip (LFOC) for micro/nanoplastics detection. (**A**) Schematic and (**B**) actual image of the LFOC. (**C**) Setup showing the chip and fiber sensor. (**D**) Real-time detection profiles of polystyrene nanoplastics (PS NPLs) at varying concentrations (0–100 μg/mL). (**E**) Linear calibration curve for 200 nm PS NPLs based on normalized signal intensity across different concentrations. Reproduced with permission from [120], Copyright 2024 Elsevier B.V.

Recent advances in microfluidic technologies have significantly improved the detection of nanoplastics in aquatic environments. These particles, typically below 1 μm in size, pose heightened risks compared to microplastics due to their increased surface area, potential for cellular uptake, and ability to cross biological barriers. Consequently, sensitive, real-time, and field-deployable detection methods are critical for effective environmental surveillance. One notable development is a laser-backscattered fiber-embedded optofluidic chip, which enables rapid, reagent-free, and non-destructive detection of nanoplastics across multiple polymer types including polystyrene, polyethylene, polyethylene terephthalate, polymethylmethacrylate, and polypropylene (Figure 7) [120]. The device achieved a low detection limit of 0.23 μg/mL and could distinguish particle sizes through a simple membrane filtration step. It demonstrated strong selectivity and operational reliability even in complex water matrices, highlighting its suitability for real-world applications. In parallel, an optical manipulation and surface-enhanced Raman scattering platform has been developed for precise nanoplastic characterization [144]. The system used gold nanoparticle stacks of varying diameters—20 μm for single-particle manipulation and 80 μm for bulk enrichment—alongside optical tweezers and Raman spectroscopy. A pre-detection cleaning step was introduced to remove natural organic matter, enhancing detection specificity. The method enabled high enrichment recovery (up to 94.3%) and extremely low detection limits (as low as 150 ng/L), allowing nanoplastics to be quantified from minimal sample volumes collected from rivers, mariculture environments, and beach runoff. Together, these microfluidic and optofluidic platforms represent a major advancement in nanoplastic detection. They offer robust tools for environmental monitoring and public health risk assessment.

#### 3.2.5. Per- and Polyfluoroalkyl Substances (PFAS)

Per- and polyfluoroalkyl substances (PFAS) are a class of synthetic compounds known for their exceptional chemical stability and resistance to degradation. Commonly referred to as “forever chemicals,” they are extensively used in industrial applications and consumer products. However, their persistence in the environment, high toxicity, and tendency to bioaccumulate have raised serious environmental and public health concerns. PFAS contamination is particularly severe in proximity to industrial sites, military facilities, and landfills, prompting an urgent need for highly sensitive and selective detection technologies.

Microfluidic platforms have recently emerged as powerful tools for PFAS detection, offering miniaturized, rapid, and field-deployable sensing capabilities. One such advancement utilized a Janus droplet-based microfluidic sensor for classifying PFAS based on their dynamic interfacial behaviors at liquid–liquid boundaries [123]. This optofluidic approach generated time-series optical emission data serving as chemical fingerprints, enabling classification of PFAS compounds by chain length and head group. The platform successfully identified four PFAS types within ten minutes, achieving 77% classification accuracy using Principal Component Analysis and Random Forest algorithms. Importantly, the method retained high performance in complex sample matrices such as synthetic groundwater and mixed PFAS solutions, demonstrating its real-world applicability.

In a complementary approach, an electrochemical microfluidic sensor was developed for detecting perfluorooctanesulfonate (PFOS), one of the most toxic and prevalent PFAS compounds [124]. Cheng et al. engineered an affinity-based platform employing a mesoporous metal–organic framework (Cr-MIL-101) that interacts strongly with both the fluorinated tail and sulfonate head of PFOS. Integrated within a microfluidic channel and positioned between interdigitated microelectrodes, this sensor achieved an ultra-low detection limit of 0.5 ng/L, surpassing U.S. EPA guidelines. Its high signal transduction efficiency and matrix tolerance allowed for rapid, high-frequency measurements in environmental samples such as groundwater. Notably, the platform also demonstrated adaptability to short-chain PFAS variants like GenX, underscoring its potential for broad-spectrum PFAS surveillance and future scalability to other hazardous contaminants.

Together, these optical and electrochemical microfluidic strategies highlight the growing potential of lab-on-chip platforms in addressing the global challenge of PFAS detection, combining sensitivity, specificity, and environmental robustness.

## 4. Integration of Microfluidic Sensors with Emerging IT Technologies

### 4.1. Smartphone-Based Platforms

Smartphone-integrated microfluidic sensors are increasingly revolutionizing point-of-care environmental diagnostics by enabling low-cost, user-friendly, and highly portable detection platforms. Smartphones offer significant advantages, including global accessibility, advanced imaging systems, on-board computational power, and wireless data transmission, making them ideal for on-site signal acquisition, real-time analysis, and remote data sharing. Multiple traditional sensing modalities—including colorimetric, fluorescent, electrochemical, and chemiluminescent methods—have been successfully miniaturized and integrated with smartphone platforms for detecting diverse environmental pollutants such as heavy metals, pesticides, and microbial pathogens. A notable example is the development of a mobile phone-integrated flow cytometer, which demonstrated the feasibility of portable and low-cost microfluidic cytometry for scalable environmental diagnostics [145]. Moreover, the integration of custom smartphone apps and optical modules has further enhanced sensitivity, quantification accuracy, and user interactivity. These innovations have significantly improved the field-readiness of smartphone-based microfluidic systems, making them increasingly viable for widespread deployment in resource-limited and remote environmental monitoring scenarios.

Recent innovations have underscored the strong potential of smartphone-integrated microfluidic biosensors for low-cost, field-ready pesticide detection, where smartphones serve as both optical readers and data processors. These systems leverage smartphone cameras and apps to enable real-time, quantitative analysis without the need for bulky instrumentation. In one study, a cost-effective three-layered paper-based microfluidic chip was developed and combined with a smartphone [146]. This platform utilized an enzyme inhibition-based colorimetric reaction for detecting profenofos and methomyl. It enabled quantitative analysis through smartphone imaging. The chip achieved detection limits of 55 nM for profenofos and 34 nM for methomyl, with a total fabrication cost of only 0.082 ¥ per piece. In another study, a microfluidic paper-based analytical device (μPAD) was integrated with a smartphone for the label-free colorimetric detection of imidacloprid and carbendazim [106]. The device employed polystyrene beads decorated with gold nanoparticles, functionalized with specific aptamers. Detection limits reached 3.12 ppm for imidacloprid and 1.56 ppm for carbendazim. The aptamers showed high selectivity against other pesticides such as thiamethoxam, fenamiphos, isoproturon, and atrazine. However, some cross-reactivity with linuron was noted. These studies demonstrate the feasibility of integrating smartphones with microfluidic devices for low-cost, portable pesticide detection, though challenges in specificity remain.

A novel microfluidic detection platform has been developed for the rapid and low-cost measurement of methylparaben (MP), a common personal care product (PCP) contaminant, in commercial food samples [147]. The system integrated a finger pump microchip (FPM) with a WiFi-enabled smartphone-based analytical setup. A small volume of sample (5 μL) underwent a modified Fenton reaction on the microchip at 40 °C to produce a green-colored complex. The resulting color intensity was analyzed using custom self-written RGB analysis software installed on a smartphone. The combined red and green (R + G) intensity values showed a strong linear correlation (R^2^ = 0.9944) with MP concentrations ranging from 100 to 3000 ppm. The platform demonstrated accurate detection in 12 commercial food samples, with deviations within 5.88% compared to standard benchtop HPLC methods. The entire analysis process was completed within 5 min. The results highlight the capability of compact microfluidic systems, paired with smartphone-based analysis, to provide fast and accurate on-site detection of PCP contaminants.

**Figure 8:**
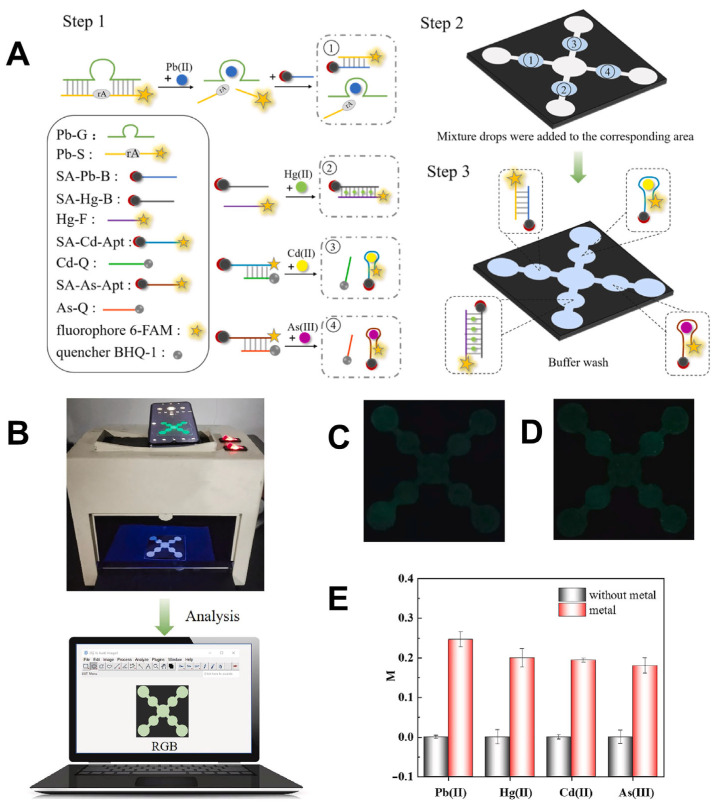
Multi-channel fluorescent paper-based chip for simultaneous metal ion detection. (**A**) Schematic of the detection system. (**B**) Image of the detection device with the paper-based chip. Fluorescence images captured by smartphone (**C**) without and (**D**) with target ions. (**E**) Comparison of M values with and without 200 nM metal ions. Reproduced with permission from [148], Copyright 2023 Elsevier B.V.

A notable example of this integration is the development of a self-driven paper-based microfluidic device (μPAD) for the simultaneous detection of four heavy metal ions: Pb^2+^, Hg^2+^, Cd^2+^, and As^3+^ in food samples (Figure 8) [148]. This μPAD employed wax and screen-printing techniques to construct capillary channels on filter paper, eliminating the need for external pumps and simplifying field use. Detection was achieved through “turn-on” aptamer-based fluorescence sensing, with results captured via smartphone imaging. The analysis was performed using RGB values extracted through ImageJ software, enabling quantitative evaluation. The device demonstrated excellent sensitivity with detection limits ranging from 1.65 to 4.20 nM and recovery rates between 84% and 104% in real samples such as apples and lettuce. This approach confirms that smartphone-enabled μPADs can serve as reliable tools for multi-target heavy metal detection in food, with strong potential for field-based applications.

Smartphone integration has proven to be a powerful strategy for enhancing the portability, affordability, and usability of microfluidic sensors. By leveraging smartphone cameras and custom analysis apps, these platforms enable rapid, on-site detection of diverse contaminants—from pesticides to heavy metals and personal care products—with high sensitivity and accuracy. This approach transforms simple microfluidic devices into smart, user-friendly tools that hold great promise for decentralized environmental monitoring and food safety applications.

### 4.2. Artificial Intelligence and Machine Learning

The integration of artificial intelligence (AI) and machine learning (ML) with microfluidic sensors is emerging as a transformative approach for advancing environmental monitoring and diagnostics. This convergence aims to enhance the analysis of large, complex datasets generated by these sensors and to improve the accuracy of environmental and health diagnostics. AI algorithms enable the identification of subtle patterns and the prediction of environmental conditions or potential health risks. Concurrently, ML techniques can optimize sensor design, increase operational efficiency, and support more effective data management and interpretation. Additionally, AI can automate experimental workflows associated with microfluidic sensors, facilitating faster, data-driven decision-making in both environmental and public health applications. Ultimately, embedding AI within microfluidic platforms paves the way for intelligent, autonomous monitoring systems capable of sophisticated pattern recognition, real-time diagnostics, and accurate pollutant detection across diverse environments.

A robust and accessible strategy for real-time, on-site detection of heavy metals—particularly mercury (Hg^2+^)—in marine environments has been achieved through the integration of microfluidic biosensors with machine learning and smartphone technologies [116]. Pennacchio et al. presented a novel, portable biosensing platform based on a hydrophobin-based chimera (Vmh2-H3w) that adhered to polystyrene plates and exhibited fluorescence quenching upon binding Hg^2+^. The sensor demonstrated high specificity and sensitivity, detecting mercury at concentrations as low as 0.3 nM in seawater and 0.4 nM in tap water, even in the presence of other metal ions. By coupling the biosensor with machine learning algorithms and using smartphone-based fluorescence image capture, the system enabled real-time prediction of mercury concentrations without the need for complex instrumentation or specialized personnel. This study underscores the critical role of machine learning in augmenting microfluidic biosensor performance, facilitating intelligent data analysis and advancing the development of scalable, user-friendly environmental monitoring systems.

The fusion of machine learning techniques with sustainable microfluidic SERS platforms provided an effective solution for real-time pesticide detection in aquatic environments [149]. A recent study introduced a sustainable, low-cost SERS substrate fabricated on paper using *Cedrus libani* plant extract, enabling the formation of silver nanoparticles (~41 nm) that significantly enhanced Raman signals. The system effectively detected four pesticides—myclobutanil, phosmet, thiram, and abamectin—despite their complex spectral profiles. To overcome this complexity, machine learning, specifically the k-nearest neighbors (k-NN) algorithm, was employed, achieving a high classification accuracy of 92.46%. This integration of green nanotechnology and machine learning enables the development of portable, smartphone-compatible microfluidic devices that deliver on-site, label-free, and highly accurate environmental monitoring through intelligent data analysis.

**Figure 9:**
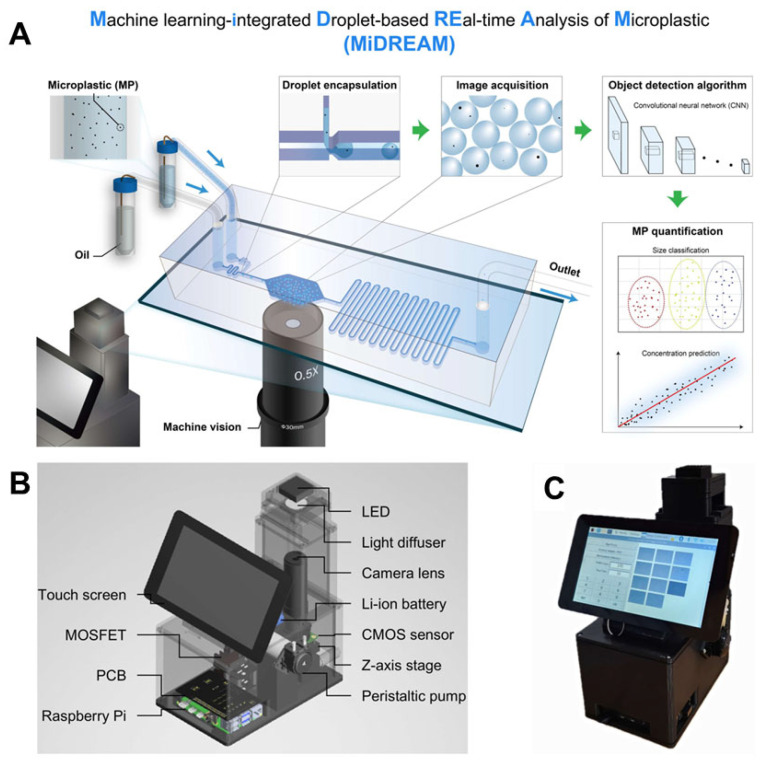
Overview of the MiDREAM system for microplastic analysis. (**A**) Workflow illustrating MP encapsulation, image acquisition via CMOS sensor, and CNN-based detection for quantification and sizing. (**B**) Structural layout of the system. (**C**) Image of the fabricated MiDREAM prototype. (**A**–**C**): Reproduced with permission from [121], Copyright 2025 Elsevier B.V.

Building upon this integration, recent advances in microfluidics have demonstrated the practical application of ML-based approaches in enhancing plastic detection capabilities, offering enhanced sensitivity, automation, and real-time analysis. For microplastic detection, the MiDREAM system (Machine learning-Integrated Droplet-based real-time Analysis of Microplastics) exemplified this progress (Figure 9) [121]. It combined high-throughput droplet microfluidics with a YOLO v8 deep learning model and a high-resolution CMOS sensor to detect and classify microplastics (3–50 μm) with exceptional accuracy (85.3–95.4%) and a mean average precision (mAP) of 0.982. The platform operated robustly across various environmental samples (e.g., DI, river, sea water) and matched the performance of established methods such as SERS and hemocytometer counts (R^2^ = 0.9965). In parallel, the detection of nanoplastics (NPs)—a more technically demanding task due to their minute size and complex environmental interactions—was addressed through an agarose-based microfiltration microfluidic device integrated with CNN-assisted Raman spectroscopy [122]. This device employed a 1% agarose matrix and micropost architecture to achieve dual-mode filtration, physical preconcentration, and high Raman signal clarity upon dehydration. It demonstrated up to 80% capture efficiency in seawater and successfully detected PSNPs at low concentrations (6.25 µg/mL), with CNN integration reducing spectral mapping time by 50%. Collectively, these innovations highlight the synergistic power of microfluidics and AI in tackling plastic pollution at both micro- and nano-scales, thereby laying the foundation for scalable, portable, and field-deployable detection platforms. Future developments should focus on increasing sensitivity to environmentally relevant concentrations (<1 µg/L), expanding detection libraries to diverse plastic types, and integrating signal enhancement techniques such as surface-enhanced Raman scattering (SERS) to further advance environmental monitoring capabilities. As plastic pollution continues to threaten ecosystems and human health, such integrated microfluidic technologies represent a critical step toward sustainable environmental diagnostics and mitigation strategies.

## 5. Conclusions and Outlook

Recent advancements in microfluidic sensors and biosensors have positioned them as promising tools for the sensitive and selective detection of micropollutants across various environmental matrices. Innovations in materials science have led to the development of biocompatible and functional materials tailored for microfluidic device fabrication, while sophisticated microfabrication techniques have enabled the creation of intricate and efficient device architectures. The integration of novel nanomaterials and biomolecular receptors has significantly enhanced the performance characteristics of these sensors, particularly in terms of their sensitivity and specificity towards target analytes. Furthermore, the development of diverse platform types, ranging from highly integrated lab-on-a-chip systems capable of performing complex analyses to highly portable devices designed for on-site use, has greatly expanded the scope of their potential applications in environmental analysis. The seamless incorporation of smartphone technologies and AI has further improved the functionality and accessibility of these systems. Together, these innovations are paving the way for next-generation environmental diagnostics: smart, autonomous, and user-friendly sensing platforms capable of real-time, on-site monitoring, even in resource-limited settings.

Despite these advances, several challenges must be addressed to enable the widespread adoption of microfluidic sensors and biosensors in routine environmental monitoring. First, scalable and low-cost manufacturing methods—particularly for devices with precise microfabrication or fragile biological components—are lacking [150]. Long-term operational stability under varying environmental conditions—such as temperature, pH, salinity, and the presence of interfering substances—poses a significant reliability issue. Biofouling, a common problem in aquatic deployments, leads to sensor surface degradation and can severely impair performance. Many platforms still rely on external equipment or trained personnel, limiting usability in remote or resource-limited settings [151]. Additionally, the lack of standardized testing protocols, performance benchmarks, and regulatory frameworks complicates validation and large-scale implementation [152].

Looking ahead, the future of microfluidic sensing technologies in environmental and public health monitoring is promising. Their potential applications are vast, ranging from enabling continuous, real-time monitoring of water and air quality to facilitating the early detection of waterborne pathogens and the assessment of environmental health risks. The integration of AI and ML for automated data analysis and decision support will further amplify their impact. Realizing this potential will require continued interdisciplinary collaboration across microfluidics, materials science, nanotechnology, biosensing, environmental engineering, and AI, laying the groundwork for transformative progress in micropollutant detection and environmental diagnostics.
